# Do Blood Lactate Levels Affect the Kinematic Patterns of Jump Shots in Handball?

**DOI:** 10.3390/ijerph182010809

**Published:** 2021-10-14

**Authors:** Ivan Belcic, Sasa Rodić, Vedran Dukarić, Tomislav Rupčić, Damir Knjaz

**Affiliations:** 1 Faculty of Kinesiology, The Laboratory for Sports Games, University of Zagreb, 10000 Zagreb, Croatia; sasa.rodic@kif.hr (S.R.); vedran.dukaric@kif.hr (V.D.); tomislav.rupcic@kif.hr (T.R.); damir.knjaz@kif.hr (D.K.); 2 High School Jastrebarsko, 10450 Jastrebarsko, Croatia

**Keywords:** movement analysis, performance, handball shot, internal load, shot precision

## Abstract

The aim of this study was to determine whether the dynamic motor stereotype of movement (shooting technique) is violated under conditions of an increased lactate concentration in a player’s blood after a 30–15 intermittent fitness test. The hypotheses was that there would be statistically significant differences in ball speed and shooting accuracy in jump shots on the goal before and after the occurrence of fatigue in the player. The sample of respondents consisted of 10 top-level handball players of the highest competition rank in Croatia. The results showed significant differences before and after the fatigue protocol in the run-up speed (*F* = 5.66; *p* = 0.02), in the maximum speed of the forearm (*F* = 5.85; *p* = 0.02) and the hand (*F* = 4.01; *p* = 0.04), in the speed in the shoulder joint (*F* = 5.39; *p* = 0.02) and wrist joint (*F* = 4.06; *p* = 0.04), and in the ball shooting speed (*F* = 5.42; *p* = 0.02). The accuracy of the shot was, on average, lower (36.20 vs. 33.17 cm) but not significantly so. High blood lactate levels affect changes in certain kinematic parameters during the performance of a jump shot in handball. Consequently, this reduces the speed of the shot, which can affect situational performance as one of the two significant parameters of scoring success.

## 1. Introduction

The evolution of handball is leading handball in the direction of an even faster and more dynamic game in which speed, agility, speed endurance, and explosive power are increasingly important for success [1,2,3]. Under these conditions, in order to achieve a logical objective that leads to victory (i.e., scoring more goals than the opponent) [4,5], attackers try to obtain and ensure the optimal position for a shooter. This is achieved by using fast movements on the field at short distances with the help of fast and strong changes in movement direction of the body, with or without the ball, using different offensive actions [6]. Handball is a physiologically demanding game that places medium-to-high demands on a player’s aerobic system, while also placing significant loads on the anaerobic energy system [7,8]. In a study conducted by Chelly et al. [9] on a sample of 18 handball players, national team members of thean average age of 15.1 years, it was found that the average heart rate was 172 ± 2 beats per minute and the average blood lactate concentration is 9.7 ± 1.1 mmol/L during the monitored championship matches. This indicates extremely demanding physiological loads during a match, in which aerobic and anaerobic modes of operation alternate.

Force production, coordination of movement, motor control precision, time of muscle reactions, and proprioception are directly negatively affected by the occurrence of fatigue [10]. Considering research on the influence of fatigue on changes in certain kinematic parameters in various specific motor movements, research in the field of football, basketball, and baseball is in the lead. Previous studies in these sports have proven certain changes in the observed kinematic parameters due to higher physiological load [11,12,13]. Cortes et al. [14] found, in a sample of football players, that physiological load significantly affects the kinematic performance of the two-foot jump from running approach and sidestep-cutting task, as seen from the increase in knee and hip extension in pre- and post-load performance. In a study of the effects of a specific exercise imitating a 90-minute football match, a group of authors [15] found that increased physiological load reduced the shot accuracy by 25%, while passing accuracy did not decrease significantly, but passing speed decreased by 7.8%. 

Shooting on the goal is considered one of the most important technical skills in competitive handball, because it is the main determinant of all actions that players take during the game [16]. Thus, a jump shot is the most common shooting technique in handball, with a total of approximately 74% of all shots during a game [4]. The kinematic parameters of the specific motor skills of shooting in the field of handball have been observed for many years. These parameters have been observed by various systems for monitoring the performance of shot movements. Much of the research is devoted to the analysis of the kinematic parameters of ground and jump shot performance and determining model values, as well as analysis of different shots on the goal [4,17,18,19,20]. A small amount of research can be found in the literature related to the effects of fatigue on the performance of the kinematic chain of ball shooting in handball. Akyuz et al. [21] researched if skeletal muscle fatigue affects shooting accuracy and ball speed in handball and found no significant differences. The authors used the following criteria for exhaustion: Maximum heart rate, plateau in VO_2_max, and a blood lactate concentration over 8 mmol/L. Thorlund et al. [22] determined the influence of fatigue on the mechanical properties of muscles and their neuromuscular activity on a sample of 10 top-level handball players during a simulated handball match. They found that reducing the ability to exert maximum force has a negative impact on performing fast movements such as acceleration, sprints, and lateral movements. In the situational conditions of a handball game, the reduced height of the jump affects the reduction in the possibility of blocking the opponent’s shot, but also the shot over the block. The effect of local fatigue on the performance of upper extremities in handball jump shots was researched by Plummer and Gretchen [23]. No significant differences in kinematic performance were found in the observed shoulder and elbow segments before and after the fatiguing protocol with the local load caused by throwing medicine balls. The authors hypothesized that the fatigue protocol loading the entire kinematic chain could cause significant changes in the shooting technique. This was confirmed by another study, which determined the effects on the kinematics of the jump shot after the functional load by a progressive load test on the treadmill [24]. Although no significant changes in performance were found before and after the load protocol, there are still visible differences after the load protocol in the angular positions of the hip and torso segments during the jump shot.

The aim of this research was to determine whether the dynamic motor stereotype of movement, i.e., the shooting technique, is violated under the conditions of increased lactate concentration in the player’s blood after a 30–15 intermittent fitness test, with hypotheses that there are significant differences in ball speed and shooting accuracy in jump shots on the goal before and after the influence of fatigue on the player.

## 2. Materials and Methods

### 2.1. Confirmation of the Ethics Committee

This research, undertaken in accordance with the Declaration of Helsinki, was approved by the Ethics Committee of the Faculty of Kinesiology, University of Zagreb. Prior to the start of the measurements, the respondents were given detailed information about the measurement protocol, the benefits, and the risks of the research. Moreover, upon arrival at the measurement venue, all respondents signed a consent form to participate in the research and to the use of their personal data.

### 2.2. Sample of Respondents 

The sample of respondents (Table 1) consisted of 10 top-level handball players of the Premier Croatian Handball League (the highest-ranking competition in Croatia). The criterion for inclusion in the research was the level of playing, i.e., the players had to be members of the highest competition rank in the Republic of Croatia. The respondents did not have any health problems in the past year, which was the most important criterion for participation in the study. To avoid the impact of fatigue on the results of measurements, the respondents had a reduced training volume and intensity and did not play any matches two days prior to the testing. Additionally, the respondents were asked and warned not to take any stimulant before testing.

### 2.3. Measurement Protocol

#### 2.3.1. Materials 

A Seca 213 portable stadiometer was used to measure body height, and a TANITA BC-545n (Bioelectrical Impedance Analysis) scale was used to measure weight and fat mass percentage. Heart rate (HR) was measured with a heart rate monitor (Polar H10, manufacturer: Polar, Kempele, Finland). Blood lactate level was measured with a portable lactate meter (Lactate Scout 3, manufacturer: SensLab GmbH, Leipzig, Germany). A blood sample was taken from the fingertip of the ring finger. Before taking the blood sample, the finger was cleaned with antiseptic solution and wiped with a liner. After puncture with a lancet, the first two drops of blood were discard and the third was used for analysis. The blood sample was taken with Lactate Scout SENSOR (EKF diagnostics, Senslab, Code 14) and inserted into a portable lactate meter. The measurement of kinematic parameters was performed with the XSENS Awinda system for kinematic analysis. This system consisted of 17 wireless sensors attached with Velcro straps to subjects body (foot, lower leg, upper leg, pelvis, sternum, shoulder blade, upper arm, forearm, hand, and head). The Awinda wireless system records with a 60 Hz frequency, a 30 ms latency, and a 1000 Hz internal sampling rate. The MVN BIOMECH software package (MVN Studio 4.4, firmware version 4.3.1) was used to analyze the data from the kinematic suit. Previous research has determined the metric characteristics and the possibility of applying this system in sports games [25,26]. The speed of the ball (IHF Official ball size 3) was measured by a speed radar (Stalker ATS II, manufacturer: Stalker Sport, Texas, USA). A progressive discontinuous load test 30–15 [27] to failure was used for the fatigue protocol. The test was interrupted when the respondent failed to reach the 3 m zone three times in a row. Upon completion of the fatigue protocol, the respondents rated the subjective feeling of load by using a modified scale [28] from 0 to 10 degrees, known as the rating of perceived exertion (RPE). The shot was filmed with a high-speed camera (Panasonic GH5, 180 FPS). Video analysis was performed using the Kinovea (v.0.8.15) software package. Video analysis provided information about ball distance from goal frame, which was used to determine the shot accuracy. Previous research [5] has justified the use of this method for measuring accuracy.

#### 2.3.2. Experimental Procedure Timeline

**Figure 1:**
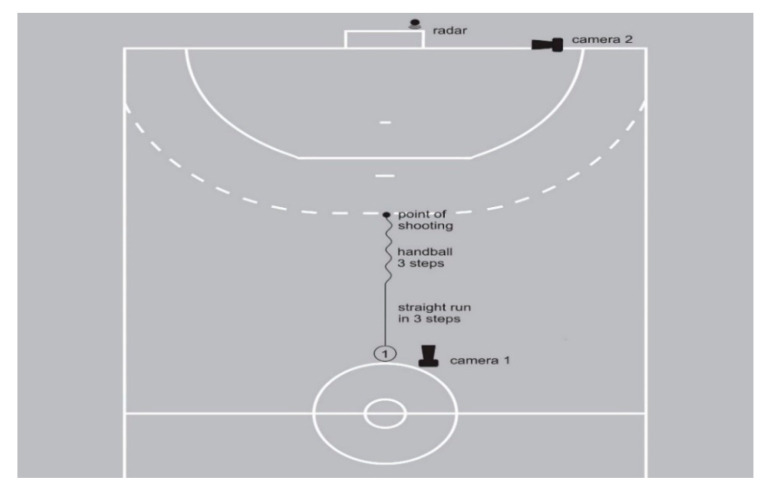
Sketch of the workplace.

**Figure 2:**
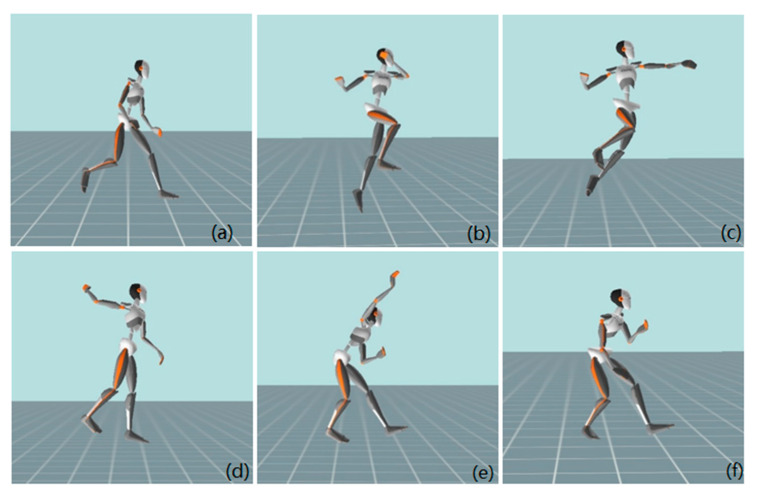
Kinogram of the final phase of the jump shot on the goal (**a**) final step; (**b**) take-off, (**c**) and (**d**) preparation for the shot; (**e**) shot, and (**f**) landing.

The measurement protocol included measuring anthropometric characteristics, warm-up, pre-load protocol measurement, fatigue test, and post-load protocol measurement. Upon arrival at the sports hall, the respondents’ height, weight, foot length, ankle height, knee height, leg length, pelvis width, shoulder height, shoulder width, and arm span were measured. These basic anthropometric characteristics were needed to describe the respondent sample and to calibrate the system for measuring kinematic parameters (Xsens technologies BV, Netherlands). Upon arriving at the sports hall, the respondents were asked to lay down on a mat and stay calm for 5 min, and resting heart rate was measured. Then, the data on lactate concentration were taken and measurement of the anthropometric characteristics was carried out. After this, the respondents began to warm up. The warm-up protocol lasted 15 min and consisted of running with tasks, dynamic warm-up with the ball, passing the ball in pairs, and 10 shots on the goal with a progressive increase in speed and strength of the shot. After the warm-up, the respondent started with the first series of (5) shots on the goal (Figure 1 and Figure 2). Between each shot was a 30 s difference, which allowed subjects to return to the starting position and prepare for the next run-up and shot. Right-handed subjects were asked to aim at the right top corner, and left-handed subjects to aim at the top left corner of the goal. The lactate concentration was then measured again, after which the respondent proceeded with the fatigue protocol. Immediately upon completion of the test, the lactate concentration was measured again, and the respondents began a second series of (5) shots.

### 2.4. Variables

In the run-up phase, the variable of maximum pelvis velocity (Pelvis_Vmax) (m/s) was observed. In the take-off phase, the variables of the lower extremities (take-off foot) were observed: Maximum angular velocity in the ankle joint (Ankle_AVmax) (°/s), maximum angular velocity at the knee joint (Knee_ AVmax) (°/s), and maximum angular velocity at the hip joint (Hip_AVmax) (°/s). In the ball shooting phase, the parameters of the dominant side of the body (the shooter’s dominant arm) were observed: Maximum shoulder speed (Shoulder_ Vmax) (m/s), maximum upper arm speed (Upper_arm_ Vmax), (m/s) maximum forearm speed (Forearm_ Vmax) (m/s), maximum hand speed (Hand_ Vmax) (m/s), maximum angular velocity at the shoulder joint (Shoulder_ AVmax) (°/s), maximum angular velocity at the elbow joint (Elbow_ AVmax) (°/s), and maximum angular velocity of the wrist joint (Wrist_ AVmax) (°/s). After the ball shooting phase, the speed of the ball (Ball_ Vmax) (km/h) and the accuracy of the shot (ACCURACY) (cm) were measured.

### 2.5. Data Processing Methods

G* power analysis calculated the total (*N* = 66) sample (number of shots) required to conduct the study with an error of *p* < 0.05, a statistical power of 0.8, an effect size of 0.25, and two groups. The Statistica v.13.05.0.17 (TIBCO software Inc) software package was used for statistical data processing. Basic descriptive parameters (mean, minimum, maximum, and standard deviation) were calculated for all observed variables. Multivariate analysis of variance (MANOVA) was used to test the statistical significance of the kinematic parameters of the shot before and after the fatigue protocol. Additionally, to test significant differences between each observed parameter, ANOVA for repeated measurements was used. A total of 100 shots were measured, of which 92 (46 before and 46 after the fatigue protocol) were used for statistical analysis. Due to errors in the performance of the motor movement (shooting technique) of the jump shot on the goal, eight shots were not analyzed.

## 3. Results

Prior to warm-up, the players were measured (with basic descriptive parameters in Table 2) for lactate concentration parameters (1.3 ± 0.52 mmol/L) and resting heart rate (83.70 ± 7.81 bpm). Moreover, the maximum heart rate was measured during the execution of shots before the fatigue protocol (163.00 ± 10.91 bpm), then during (195.40 ± 8.30 bpm) and after the fatigue protocol (171.40 ± 7.04 bpm) during the execution of shots under physiological load. The level of blood lactate concentration was measured after the first shots (2.07 ± 1.16 mmol/L) and after the fatigue protocol (11.88 ± 3.33 mmol/L). The maximum heart rate was 204 bpm, and the lactate concentration was 18.4 mmol/L. During the 30–15 fatigue protocol test, the respondents achieved an average running speed of 18.20 ± 1.01 km/h. The minimum running speed was 16 km/h, while the maximum was 20 km/h. The fatigue test was rated, on average, as very severe activity (8.2 scores on a modified Foster subjective feeling scale [29]).

Table 3 shows the basic descriptive indicators and results of the ANOVA between the kinematic parameters of the shot before and after the fatigue protocol. The position of the hand during the performance of the jump shot was lower (2.18 cm) after the exhaustion protocol. The highest position of the shooter’s hand was not significant (*p* = 0.39). The speed of the run-up also decreased under the influence of fatigue (before, 5.45 m/s; after, 5.26 m/s). The difference in the reduction of the speed of the running start was significant (*F* = 5.66; *p* = 0.02). In the ball shooting phase, the angular velocities of the segments (shoulder, upper arm, forearm, and hand) were, on average, slower after the fatigue protocol. The highest maximum velocity in the ball shooting phase was reached in the wrist before the fatigue protocol (16.05 m/s). At this stage, a significant difference was obtained in the maximum velocity of the forearm (*F* = 5.85; *p* = 0.02) and the hand (*F* = 4.01; *p* = 0.04). Moreover, the angular velocities (shoulder, elbow, and wrist) were higher prior to performing the fatigue test. The highest angular velocity was reached at the shoulder joint (2424.74 °/s). Significant differences were obtained in the shoulder joint (*F* = 5.39; *p* = 0.02) and wrist (*F* = 4.06; *p* = 0.04), while in the elbow joint, there was no difference between the observed groups. During the take-off phase, the mean values of angular velocities in the hip and knee joint were higher before the fatigue protocol, while in the ankle joint, they were higher after the protocol. The maximum angular velocity was reached in the ankle joint (1010.68 °/s). No significant differences between groups were obtained in the take-off phase. The speed of the ball differed significantly under the influence of fatigue (*F* = 5.42; *p* = 0.02). After the fatigue protocol, the speed decreased by an average of 2.76 km/h. The maximum achieved shot speed was 97.70 km/h, while the lowest was 68.60 km/h. The shot accuracy was, on average, lower (36.20 vs. 33.17 cm) after the fatigue protocol, but no significant difference was obtained.

## 4. Discussion

The aim of this study was to determine whether the dynamic motor stereotype of movement (shooting technique), i.e., the ball shooting speed and the accuracy of shooting, is violated during jump shots on the goal under conditions of an increased lactate concentration in the player’s blood after the test. Significant differences in the kinematic parameters of the shot on the goal before and after the fatigue protocol prove a change in the complete kinematic chain of technique, i.e., motor knowledge of shooting on the goal. This is particularly true for the dominant upper extremities, where a significant difference was visible (at maximum forearm and hand speed), which also influences the speed of a shot.

The speed of running and the realization of the spatial–temporal advantage of the start and the start acceleration, i.e., the first step, is extremely important for the situational success in handball, both individual and team. Counterattack is the main determining factor of success in teams of the same rank in handball [30] and the most efficient method of scoring a goal with 88.23% of success rate. Accordingly, the change of rules in handball resulted in the concentration of the best teams on counterattack tactics (and also speeding up the game during the attack). The reaction time and the players’ speed are the factors that allow them to gain an advantage over an opponent in the counterattack stage of the game, which has an impact on the final score. Consequently, the players’ speed leads to easier realization, a better position to shoot on the goal, and a better position to pass to a player who is in a better position. Meanwhile, reducing speed during the fatigue phase reduces the situational efficiency of the individual player and the team. After the 30–15 test, aimed at fatiguing the players and achieving high blood lactates levels, a significant difference in the running approach speed (best performance speed for jump shot) was obtained (*p* = 0.02). Only one player had a blood lactate level below 8 mmol/L, which was the reference value used in a similar study [21], where the authors tested if skeletal muscle fatigue affects shooting accuracy and ball speed in handball. By observing the speed parameters, these findings indicate a reduced individual situational efficiency and player abilities. Achieving the spatial–temporal advantage with regard to the running start speed and running was reduced in the fatigue phase. This consequently reduced the success of passing in a one-on-one game, in fast phases of the game such as fast transition, and individual breakthrough or counterattack, which is the fastest way to score a goal on an opposing team, since it does not have enough time to organize the defensive phase. Moreover, the reduced speed of the body affected the final speed of the shot, because the higher the speed of the body during the shot, the higher the speed of the shot on the goal [16,20].

The average values of the maximum velocity of the upper body segments that play a key role in the ball shooting phase (shoulder, upper arm, forearm, and hand), looking at the average values achieved in the tests, were reduced after the fatigue protocol. This was most pronounced in the significant differences before and after the protocol, where the velocities of the forearm (*p* = 0.02) and the hand (*p* = 0.04) were reduced after the fatigue protocol. A reduced speed of the forearm and hand has a negative effect on the speed of the shot, because they are the last parts of the body that are activated in the final phase of the shot on the goal [29]. Moreover, depending on the shooting technique (whether there is more emphasis on the wrist), a reduced speed in certain parts of the body such as the hand and forearm can vary. Herein, the influence of physiological load on changes in the observed kinematic parameters of the upper extremities was not frequently observed. However, Tripp et al. [31] observed the influence of physiological load on the positions of the joints in charge of throwing a baseball, which has similar biomechanical principles in throws to a handball shot and found that the sensorimotor system is impaired under the influence of fatigue. Uygur et al. [32] analyzed the effects of fatigue on the kinematics of free throws in basketball, where the respondents were subjected to a fatigue protocol with physiological load. They found that the maximum physiological load provoked by running sprints up to 30 m and two-foot jumps until failure in top-level seniors does not influence the kinematic parameters of performance or accuracy. On the contrary, an impact on performance in younger basketball players was visible.

The angular velocities of the kinematic chain (shoulder, elbow, and wrist) were higher before the fatigue test, and significant differences were obtained at the maximum angular velocity in the shoulder joint (*p* = 0.02) and the wrist (*p* = 0.04). As a result of the stated reductions in the speed of the shoulder and wrist joints, and the reduction of the speed of the hand and forearm after the fatigue protocol, a significant difference in the speed of shooting on the goal was obtained. This is a logical sequence due to a reduction in the maximum speed in almost all parameters that affect the extension of the elbow, which is a determining factor for shooting speed [33,34]. In team handball, the ability to score largely depends on the ball speed and the accuracy of shoots. A quick shot directed toward the goal is considered an advantage to beat the goalkeeper and score a goal, which is the main objective of the game. This is the reason why coaches concentrate on training to increase shooting speed with an emphasis on optimizing shooting technique [35]. The obtained results indicate a decrease in the shot speed, which is one of the two significant abilities for scoring, while the shooting accuracy did not differ significantly before and after the fatigue protocol. The accuracy of the shot on the goal was reduced in the average values before and after the fatigue protocol, but it was not significant. The accuracy of the shot [36] is, in addition to the speed of the shot, one of the two parameters of success in scoring [4,5]. By reducing the accuracy and speed when shooting on the goal, the chances of successful team defense and individual goalkeeper defense increase. This is especially important under situational conditions or in positional shots, where the speed of the shot is not as significant for shooting success (a similar conclusion was obtained in a water polo study [37]). Examples include shooting from wing positions, shooting in a one-on-one situation with a goalkeeper, or shooting from a pivot’s position. The other monitored variables such as maximum shoulder and upper arm velocity in the upper body and maximum angular velocity at the elbow joint, on average, decreased after the fatigue protocol. Segments of the lower body also indicate a decrease in the maximum angular velocity in the hip and knee joint in the average values, but the abovementioned upper and lower body parts did not experience significant deviations after the 30–15 test. Of all of the variables, only the maximum angular velocity of the ankle was increased, but not significantly.

The maximum speed in the shoulder joint, especially the flexion of the shoulder, together with the extension of the elbow and the deviation of the wrist, had the greatest influence on the speed of the shot. Ulnar deviation of the joint causes a higher speed ball rotation, which positively affects the quality of the ball flight, and thus the ball speed. Top-level handball players have an individual automated movement from the preparation to the realization of the shot, i.e., a dynamic motor stereotype of shooting on the goal, which they additionally emphasize in the final phase of the shot with their wrist [38]. Thus, top-level handball players, both with the influence of the load and the change in the kinematic chain of the entire motor knowledge, still maintain the accuracy of the shot, assuming that they avoid a foul attempt in defense [7]. Due to the reduction in shot speed from the aspect of situational efficiency, it is recommended for coaches to include, in the training process (technical or specific-situational shooting trainings), contents that are carried out with a physiological load similar to handball matches. This is an important factor in the training process in order to change and facilitate later adaptations of the entire motor knowledge (the players’ movement) to fatigue that occurs during matches. Moreover, it is essential in the phases of the match when the physiological load is greatest, because it is in these phases that the winner of the match, competition, or championship is decided.

The strength of this research is reflected in the quality sample of respondents who play in the highest national rank of the competition. The research limit is manifested in the playing positions and situational conditions during the match. The speed of the shot in certain positions or situations in the game is not the most important element, but precision is, which comes to the fore (shooting from the wing position at a reduced angle or shooting from the pivot’s position under pressure or foul). However, each player used the same jump shot technique, which is used when shooting from outside positions without pressure or contact with defensive players.

## 5. Conclusions

High blood lactate levels affect changes in certain kinematic parameters during the performance of a jump shot in handball, as well as a change in the complete kinematic chain of performing the technique of shooting on the goal. This is especially pronounced in the upper extremities of the body in the form of a decrease in the speed of the forearm and hand and in the maximum angular velocities of the shoulder and wrist joints. The complete technique of shooting in handball is a sequence of successive actions of individual parts of the body. The final part of this series is the dominant arm and extension in the elbow, where the last phase ends with the forearm and hand. As a result of changes in the kinematic chain in the performance of the jump shot technique, the shot speed was reduced after the fatigue protocol. This can consequently affect situational performance, since the shot speed is one of the two significant parameters of successful scoring. Recommendations for coaches involve including technical and specific-situational shooting trainings with (high) physiological loads in their training process to simulate fatigue, which occurs in handball matches.

## Figures and Tables

**Table 1:** Basic descriptive statistics of the respondents (*n* = 10).

Variable	Valid *N*	Mean	Minimum	Maximum	Std. Dev.
Age (years)	10	19.32	18.02	21.96	1.21
Height (cm)	10	188.35	179.00	197.50	5.61
Weight (kg)	10	86.42	71.40	106.20	9.62
Fat mass (%)	10	12.44	6.60	20.90	4.15

**Table 2:** Basic descriptive parameters of the selected variables.

Variable	Valid *N*	Mean	Minimum	Maximum	Std. Dev.
R_HR	10	83.70	68.00	92.00	7.81
HR_max_1st	10	163.00	150.00	184.00	10.91
HR_max_2nd	10	171.40	158.00	179.00	7.04
30–15_HR_max	10	195.40	181.00	204.00	8.30
R_Lac	10	1.30	0.70	2.20	0.52
Lac_1st	10	2.07	0.90	4.50	1.16
30–15_Lac	10	11.88	7.20	18.40	3.33
30–15_V	10	18.20	16.50	20.00	1.01
30–15_BORG	10	8.20	7.00	9.00	0.63

Legend: RHR, resting heart rate; HR_max_1st, maximum heart rate after first set of 5 shots; HR_max_2nd, maximum heart rate after second set of 5 shots; 30–15_HR_max, maximum heart rate during the 30–15 test; R_Lac, resting blood lactate concentration; Lac_1st, blood lactate concentration after first 5 sets of shots; 30–15_Lac, blood lactate concentration after the 30–15 test protocol; 30–15_V, maximum speed reach in the 30–15 test; 30–15_BORG, subjective evaluation of exhaustion after the 30–15 test.

**Table 3:** Basic descriptive indicators and ANOVA results for repeated measurements before and after the fatigue protocol for the observed variables.

Variable	*N*	Mean	Min	Max	Std. Dev.	–95%CI	+95%CI	*F*	*p*
Hand_H_pre	46	246.32	222.93	262.70	11.37	–2.89	7.25	0.73	0.39
Hand_H_after	46	244.14	216.38	267.05	13.05				
Pelvis_V _pre	46	5.45	4.32	6.35	0.39	0.03	0.34	5.66	0.02 *
Pelvis_V _after	46	5.26	4.60	6.15	0.35				
Shoulder_V_pre	46	4.58	3.43	6.19	0.63	–0.09	0.45	1.69	0.20
Shoulder_V_after	46	4.41	3.05	6.43	0.68				
Forearm_V_pre	46	11.38	10.08	12.75	0.66	0.06	0.59	5.85	0.02 *
Forearm_V_after	46	11.06	9.82	12.47	0.61				
Upper_arm_V_pre	46	6.12	5.02	7.10	0.51	–0.13	0.30	0.65	0.42
Upper_arm_V_after	46	6.03	5.12	7.11	0.51				
Hand_V_pre	46	12.49	10.33	16.05	1.28	0.00	0.93	4.01	0.04 *
Hand_V_after	46	12.03	10.05	14.64	0.92				
Shoulder_AV_pre	46	1378.09	635.38	2424.74	489.06	32.89	421.83	5.39	0.02 *
Shoulder_AV_after	46	1150.72	323.98	2212.20	449.01				
Elbow_AV_pre	46	1163.05	458.06	1846.28	318.73	–145.12	152.94	0.00	0.96
Elbow_AV_after	46	1159.14	483.28	2092.63	396.56				
Wrist_AV_pre	46	754.91	334.31	1452.54	256.92	1.63	220.96	4.06	0.04 *
Wrist_AV_after	46	643.62	279.73	1484.94	272.33				
Ankle_AV_pre	46	687.34	373.93	965.48	147.88	–73.58	48.05	0.17	0.68
Ankle_AV_after	46	700.11	393.48	1010.68	145.75				
Hip_AV_pre	46	420.97	226.30	548.86	53.21	–14.05	24.58	0.29	0.59
Hip_AV_after	46	415.71	337.20	525.70	38.93				
Knee_AV_pre	46	550.06	381.10	696.09	72.60	–23.50	32.44	0.10	0.75
Knee_AV_after	46	545.60	401.37	723.48	62.01				
Ball_V_pre	46	87.28	69.60	97.70	5.47	0.41	5.12	5.42	0.02 *
Ball_V_after	46	84.52	68.60	93.70	5.89				
Accuracy_pre	46	36.20	-5.65	106.62	25.71	–7.96	14.02	0.30	0.59
Accuracy_after	46	33.17	0.94	118.89	27.30				

* Hand_H, highest position of the shooter’s hand; Pelvis_V, maximum pelvis speed; Scheme 0. *F* = 2.38; *p* = 0.00.

## Data Availability

Data available upon request.
